# Tissue Adhesives for Hernia Mesh Fixation: A Literature Review

**DOI:** 10.7759/cureus.10494

**Published:** 2020-09-16

**Authors:** Samira R Ibrahim, Peter J Ward

**Affiliations:** 1 Medicine, West Virginia School of Osteopathic Medicine, Lewisburg, USA; 2 Biomedical Sciences, West Virginia School of Osteopathic Medicine, Lewisburg, USA

**Keywords:** tissue adhesive, cyanoacrylate, fibrin glue, inguinal hernia, pain, hernia mesh, mesh fixation

## Abstract

In an effort to optimize the results during inguinal hernia repair, surgeons need to consider the role of different mesh fixation techniques. The use of tissue adhesives is proposed for mesh fixation, which demonstrate similar or improved outcomes in the realm of postoperative pain, hernia recurrence, cost, and formation of a hematoma or seroma. In this review, mesh fixation using fibrin glue and cyanoacrylate glue is compared to standard suture or tack techniques. The results of this investigation warrant consideration by surgeons seeking to improve patient outcomes.

## Introduction and background

Introduction

Inguinal hernia repair is one of the most common surgical operations in the world. Surgeons and patients are always striving to improve postoperative outcomes. Currently, postoperative pain is reported as the main undesirable outcome in inguinal hernia repair. It is thought that the postoperative pain following inguinal hernia repair may be the result of hernia mesh fixation methods. Current universally accepted fixation techniques include suture or tacks. It is thought these methods contribute to postoperative pain from nerve injury or tissue damage. Surgeons seek to decrease negative postoperative outcomes such as hernia recurrence, cost, and hematoma and seroma formation. 

Alternative mesh fixation techniques using tissue adhesives have demonstrated promising postoperative outcomes, leading to improved surgeon and patient satisfaction. Mesh fixation with fibrin glue or cyanoacrylate tissue adhesive as an alternative to standard suture or tack fixation has been investigated, showing similar or improved postoperative outcomes. These methods of hernia mesh fixation have still not been universally accepted. 

Fibrin glue, known as Tissel® or Tissucol® (Baxter Healthcare, Deerfield, IL, USA), is a biologic hemostatic agent consisting of human fibrinogen and thrombin. When activated by calcium chloride, it transforms into a strong fibrin fiber matrix. Cyanoacrylate, a chemical glue, is known as Dermabond® (Ethicon, Somerville, NJ, USA), SurgiSeal™ (Adhezion Biomedical, Wyomissing, PA, USA), Histoacryl® (B. Braun Medical, Melsungen, Germany), Glubran® (GEM Srl, Viareggio, Italy), and others. It is most commonly used in the context of closing small surgical wounds or traumatic lacerations. It has special bonding properties that form strong chains in the presence of moisture. 

Here we present a review of the literature regarding outcomes of postoperative pain, hernia recurrence, cost, and hematoma and seroma formation using fibrin and cyanoacrylate tissue adhesive mesh fixation. Outcomes are compared to the standard suture or tack technique. 

Materials and methods 

PubMed and GoogleScholar were used to screen for relevant sources. Search engine keywords and phrases included: fibrin glue, cyanoacrylate, inguinal hernia mesh fixation, tissue adhesives. Out of 140 results, 11 were selected: five articles analyzing fibrin glue and six articles analyzing cyanoacrylate glue. Articles were considered if their text included the following topics or met the stated criteria: inguinal hernia repair, open repair, laparoscopic totally extraperitoneal (TEP) repair, laparoscopic transabdominal preperitoneal (TAPP) repair, clinical relevance to the surgeon, publication in a peer-reviewed journal, outcomes regarding pain, hernia recurrence, cost, or hematoma and seroma formation. Inguinal hernia repair was chosen as the main focus of the review since it was shown to be the most common in vivo application of tissue adhesives.

## Review

Discussion

Overview: Pain 

Pain is regarded as the most disabling outcome following inguinal hernia repair. Tissue adhesive mesh fixation has shown promise in reducing postoperative pain. This result may be attributed to less irritating potential of adhesives, reduced nerve compression or injury, and lower tissue tension [1]. Tissue adhesives can be used in the triangle of pain regions, where tacks and sutures are prohibited [2]. Studies of postoperative pain using tissue adhesive mesh fixation demonstrate similar or improved pain outcomes compared to standard fixation techniques. These findings warrant consideration by surgeons. Larger studies using standardized pain scores, adequate pain classification by type, frequency, and intensity, and examining different hernia repair methods will produce a more consistent consensus on pain outcomes. Overall, the results indicate it is reasonable to use tissue adhesive techniques in patients more prone to pain [3]. The results are summarized in Table 1. 

**Table 1:** Postoperative Outcomes Using Fibrin and Cyanoacrylate Glue Compared to Traditional Mesh Fixation Methods NRS – Numeric Rating Scale, VAS – Visual Analog Scale

Postoperative Outcomes Using Fibrin and Cyanoacrylate Glue Compared to Traditional Mesh Fixation Methods		
	Fibrin Glue	Cyanoacrylate Glue
Pain	Lower incidence of pain at three months compared to sutures [4]. Lower NRS pain score at seven days and one year compared to sutures [1]. Lower VAS pain score at one day, seven days, and one month compared to sutures [5]. 5.9% chronic postoperative pain rate after three months, mostly mild [2]. Lower incidence of groin numbness and discomfort compared to sutures [6].	Less intense pain at day two and day seven compared to sutures [7]. Lower postoperative pain during everyday activities [8]. Less number of painful areas (abdominal, inguinal, genitofemoral) at six weeks and six months compared to spiral tacks [9]. Lower VAS pain score at 48 hours and seven days [10].
Hernia Recurrence	Minimal 1.5% hernia recurrence rate [2].	0% hernia recurrence at six months compared to 5% recurrence with no fixation [11].
Cost	Higher cost than sutures. Balanced overall cost when analgesic use, operative time, return to daily activities, hospital stay taken into consideration [4].	Lower cost than sutures, especially when return to activity and length of hospital stay taken into consideration [7,11].
Seroma and Hematoma Formation	Lower seroma and hematoma formation compared to sutures [4,5].	7.6%-9.8% rate of seroma formation, resolved using conservative methods [7,10].

Fibrin Glue: Pain

In a meta-analysis of open inguinal hernia repairs with fibrin glue fixation, data showed a higher incidence of chronic pain in the suture group compared to the fibrin glue group [4]. The fibrin glue group had a lower incidence of chronic pain at three months than the suture group (relative risk (RR) 0.42, 95% CI 0.22-0.79, p<0.01). A Numeric Rating Scale (NRS) was utilized in a study to evaluate pain on the first and seventh day following open inguinal repair [1]. The scores ranged on a spectrum of no pain to unbearable pain. The NRS score at follow-up was lower for the fibrin glue group at seven days and one year compared to the suture group, both with p<0.05. No differences were noted at six months. In another study, pain intensity in open inguinal hernia repair was quantified by Visual Analogue Scale (VAS) on a scale from 0 to 10 at one day, seven days, one month, and three months postoperatively [5]. The fibrin glue group showed significantly lower VAS scores one day, seven days, and one month after surgery compared to the suture group (mean VAS day one: 3.53 vs. 4.70, day 7: 2.23 vs. 3.16, one month: 0.80 vs. 1.56, p<0.0001 at all points). There was no statistical difference in the VAS score at three months between the groups with p=0.45. Another study collected data from TEP inguinal hernia repairs by a single surgeon using fibrin sealant method [2]. The study reported a 5.9% chronic postoperative pain rate after three months with this method. Most of these cases were mild and did not interfere with activities of daily living, with 0.5% of patients reporting moderate pain after one year. In another study of open inguinal hernia repairs, patients were examined preoperatively and one week after surgery [6]. Numbness and groin discomfort were lower using fibrin glue compared to sutures (mean numbness VAS 4.1 vs. 7.4, mean groin discomfort VAS 7.1 vs. 10.2, p=0.019, p=0.049, respectively). However, there was no significant difference in pain scores between the fibrin glue and suture group. This study also utilized a combined PND VAS score (Pain, Numbness, and Groin Discomfort) to quantify outcomes. Interestingly, preoperative PND VAS >30 was a significant predictor of one year PND in patients with suture mesh fixation. This suggests preoperative PND may be an indicator of postoperative PND in patients undergoing standard suture fixation. 

Cyanoacrylates: Pain

In a study comparing cyanoacrylate glue to standard suture in open inguinal hernia repairs, VAS pain scores on a scale of 1 to 10 favored the cyanoacrylate group until day 30 [7]. In particular, on day two and day seven, the cyanoacrylate group reported less intense pain compared to the suture group (mean day two VAS: 43 vs. 50, mean day 7 VAS: ~21 vs. ~30, p<0.001 for both). After day 30, the scores became similar. Both patient groups took similar amounts of analgesics up until hospital discharge. In a study of TAPP hernia repair with cyanoacrylate glue, patients described the occurrence of pain using a VAS score [8]. It was shown that VAS scores were lower after surgery compared to before surgery (mean VAS before surgery: 4.28, SD=2.16). After one week, mean VAS was 2.28 (SD=1.79), and after one year mean VAS was 0.38 (SD=0.35), both with p<0.001. The study also looked at postoperative pain during several everyday activities such as climbing stairs, light sport exercises, shopping, driving, and standing. Less postoperative pain while performing the everyday activities was noted in the cyanoacrylate group compared with preoperative reports. It was also revealed that patients below age 40 tend to report significantly higher preoperative VAS scores compared to middle age (age 40-60) and elderly (age 60+) patients (mean middle age pre-op/post-op VAS: 4.9 vs. 0.63, p=0.008, mean elderly pre-op/post-op VAS 3.8 vs. 0.29, p=0.014). In an analysis of postoperative pain after TAPP inguinal hernia repair, cyanoacrylate glue resulted in a decrease in the number of total painful areas (abdominal, inguinal, genitofemoral) at six weeks and six months compared to spiral tacks (26% vs. 11%, p=0.007 and 23% vs. 11%, p=0.022, respectively) [9]. However, there was no reported difference in VAS pain scores between the titanium spiral tack and cyanoacrylate group. In another study, VAS scores were obtained at 48 hours and seven days after laparoscopic TEP inguinal hernia repair [10]. By day seven, the cyanoacrylate group demonstrated statistical superiority in VAS scores compared to the suture group (mean VAS 0.41 vs. 0.74, significance not stated). 

Overview: Hernia Recurrence

Hernia recurrence is regarded as one of the rather most undesired outcomes in inguinal hernia repair. Most inguinal recurrence occurs within five years [4]. Tissue adhesives usually degrade in three to nine months, allowing time for sturdy fixation [11]. Appropriate mesh size is an important consideration in preventing recurrence. Proper mesh fixation is another important consideration in preventing recurrence. The type of surgical method used may also play a role in recurrence [2]. Hernia recurrence outcomes are comparable between tissue adhesives and standard fixation techniques. With the adhesive technique, there are fears of mesh dislocation that can result in recurrence. Adhesive strength between glues varies, with a study showing fibrin glue having an adhesive strength of 64.3 newtons and cyanoacrylates with a strength of 105.4 newtons [10]. The use of tissue adhesive may have an advantage over standard techniques in preventing recurrence by fixating the entire surface of the mesh to the surface of interest [2]. Some authors propose tissue adhesive as a sound alternative to standard techniques in patients prone to pain, at the expense of a potentially higher recurrence rate. The results are summarized in Table 1. 

Fibrin Glue: Hernia Recurrence

A minimal 1.5% hernia recurrence rate was found using fibrin sealant in TEP inguinal hernia repairs [2]. 

Cyanoacrylates: Hernia Recurrence 

Cyanoacrylate fixation demonstrated superior results compared to no fixation in TEP inguinal hernia repairs [11]. At six months, hernia recurrence was reported in 5% of control cases with no fixation and 0% of cases with cyanoacrylate fixation (p=0.043). Mitura et al. reported a minimal 0.7% hernia recurrence rate following TAPP inguinal hernia repair at 11 months with cyanoacrylate glue [8]. In this study, the recurrence was noted after a repair of a large medial hernia. 

Overview: Cost

Tissue adhesives have demonstrated a comparable cost-benefit ratio compared to standard mesh fixation techniques. Cost is a function of many variables, including operating time, hospital stay, and time missed from work during postoperative follow-up. Patients often express the acceptance of the slight cost differences when recompensed by shorter hospital stays and duration away from work [1]. The potential savings with tissue adhesives compared to standard techniques warrant consideration by surgeons. The results are summarized in Table 1. 

Fibrin Glue: Cost

In a meta-analysis of open inguinal hernia repairs with fibrin glue fixation, the individual price of the fibrin glue was found to be higher than sutures, but the overall eventual cost of the two procedures was similar [4]. The balanced overall cost was attributed to reduced analgesic use, operative time, return to daily activities, and hospital stay in the fibrin glue group. In a study of fibrin glue in open inguinal hernia repairs, it was concluded that a minor increase in costs using fibrin glue was balanced by an improvement in the quality of life; in particular, earlier return to work and less sick days [1]. It was revealed in another study that the mean surgical time was 10% less in the fibrin glue group compared to the suture group in open inguinal hernia repairs with p<0.001 [5]. 

Cyanoacrylates: Cost

Results in a study found a lower overall hospital cost in the suture group compared to the cyanoacrylate group in a study of cyanoacrylate mesh fixation for TEP inguinal hernia repair [11]. These costs included inspection, surgical, anesthesia, and equipment use fees. A study reports that in India the cost of 0.5 mL cyanoacrylate glue is similar to a foil pack of vicryl suture, while tacks are significantly more expensive [10]. Cyanoacrylate glue and vicryl suture costs approximately 6 USD (~450 rupees). Tacks, although unspecified which kind or how many, are reported to cost approximately 300 USD (~22,000 rupees). However some studies show fibrin glue to be as costly or even more costly than tacks [10]. In a study of open inguinal hernia repairs, there was a significantly reduced time required to return to normal daily activities in the cyanoacrylate group compared to the suture group [7]. The cyanoacrylate group had an average of three days and the suture group had an average of five days. In another study of open inguinal hernia repairs, it is reported that there is a reduced operation time in the cyanoacrylate group versus the suture group (mean operation time 73 vs. 79 minutes, respectively) [3].

Overview: Seroma and Hematoma Formation

There are differences among studies regarding seroma and hematoma formation in tissue adhesive mesh fixation. Seromas are commonly observed following hernia repairs and are most likely due to local inflammation, while hematomas are caused by injuries to blood vessels [1]. Generally these are mild complications and are treated using conservative methods or resolve spontaneously. It has been postulated by some surgeons that tissue adhesives may have a hemostatic effect [11]. The results are summarized in Table 1. 

Fibrin Glue: Seroma and Hematoma Formation

In a meta-analysis examining the use of fibrin glue versus suture mesh fixation, the trials reported higher hematoma and seroma formation in the suture group than the fibrin glue group (p=0.02) [4]. In another study of TEP inguinal hernia repairs, only 0.5% of subjects with fibrin sealant fixation of demonstrated seroma formation [2]. In another study of open inguinal hernia repairs, there was less seroma formation postoperatively favoring the fibrin glue group versus the suture group (p=0.033) [5]. 

Cyanoacrylates: Seroma and Hematoma Formation

In a study of open inguinal hernia repair, it was found that 9.8% of cases saw hematoma formation in the early postoperative period [7]. Although it was not specified if these patients were in the cyanoacrylate group or suture group, this mild complication was treated using conservative methods. Another study reported a total of 7.6% of patients in laparoscopic TEP inguinal hernia repair developed a local seroma [10]. Although it is unclear which group these patients belonged to, all seromas resolved spontaneously in six months. In the standard suture group one patient developed a hematoma that resolved by one year, and no hematoma developed in the cyanoacrylate group. 

## Conclusions

The results of this literature review demonstrate that mesh fixation in inguinal hernia repair using fibrin glue and cyanoacrylate glue results in statistically similar or improved overall postoperative outcomes compared to standard suture or tack fixation. Many studies demonstrate the efficacy of these novel methods, while some show no statistical superiority. The postoperative inguinal hernia repair outcomes are not only a function of the type of mesh fixation, but also a function of the type of surgical technique employed (open, TEP, TAPP). Perhaps the subcategorization of the results by surgical technique will reveal additional data. The universal acceptance of tissue adhesive techniques should be considered once the literature demonstrates more compelling evidence using larger patient sample sizes. For now, the data suggests that using tissue adhesive methods can be beneficial on a case-by-case basis, such as in those more prone to pain or those seeking early return to work. Fixation with tissue adhesives may reduce nerve and tissue injury and enhance stability at the expense of possible minor complications. In conclusion, repair of inguinal hernias using mesh fixation with fibrin glue or cyanoacrylate glue may be used as a simple, effective alternative to standard suture or tack fixation. 
